# Comparison of the Acute Physiology and Chronic Health Evaluation Score (APACHE) II with GCS in Predicting Hospital Mortality of Neurosurgical Intensive Care Unit Patients

**DOI:** 10.5539/gjhs.v4n3p179

**Published:** 2012-05-01

**Authors:** Ali Reza Zali, Amir Saied Seddighi, Afsoun Seddighi, Farzad Ashrafi

**Affiliations:** 1 Neurosurgery Research Center of Shohada Tajrish Hospital, Shahid Beheshti University of Medical Sciences, Tehran, Iran

**Keywords:** head trauma, mortality, GCS, APACHE

## Abstract

**Background:**

The Glasgow Coma Scale (GCS) is popular, simple, and reliable, and provides information about the level of consciousness in trauma patients. However, a systemic evaluation scale especially in patients with multiple traumas is so important. The revised Acute Physiology and Chronic Health Evaluation system type 2 (APACHE II) is a physiologically based system including physiological variables. This study compares the efficacy of the predicting power for mortality and functional outcome of GCS and APACHEII in patients with multiple traumas in intensive care unit.

**Methods:**

This study included the patients with head injury associated with systemic trauma admitted in the ICU of Shahid Rajaee Hospital in 2007 and 2008. Sensitivity, specificity and correct prediction of outcome by GCS and APACHE II were assessed and compared.

**Results:**

This study included 93 patients (79 males, 14 females; mean age 60.5; range 14 to 87 years) with head injury associated with systemic trauma in 2007 and 2008. Mortality increased in the elderly group. The mean survival score using APACHE II was 36.5 and death score was 67.4. These values using GCS were 10.3 and 6.8, respectively.

**Conclusion:**

For the assessment of mortality, the GCS score still provides simple, rapid and effective assessment in head injury patients, however, for the prediction of mortality in patients with multiple trauma APACHE II is superior to GCS since it includes multiple systemic parameters in these patients.

## 1. Introduction

A growing focus on health quality and mortality risk increased the need for accurate severity scoring systems in patients (Khuri, Daley, Henderson, et al., 1998). With accurate severity scales we can compare clinical outcomes. The risk stratification of trauma patients has traditionally focused on anatomic or physiologic scores specific to trauma populations. Several systems have been developed for evaluation of trauma patients and these scores have been previously reviewed (Chawda, Hildebrand, Pape, & Giannoudis, 2004). Among the most commonly used are the injury severity score (ISS) and the Trauma and Injury Severity Score (TRISS).

ISS is an anatomic scoring system developed in 1974 for the assessment of multiple trauma patients (Baker, O’Neill, Haddon, & Long, 1974).

The lack of physiologic data led rapidly to revisions of the ISS. TRISS is the revised version but also incorporates the Revised Trauma Score (RTS), the patient’s age and the mechanism of injury to determine a patient’s predicted survival (Boyd, Tolson, & Copes, 1987).

Scoring systems have been continuously developed to predict outcomes in patients with severe illness, to improve resource allocation and to assist in clinical decision-making particularly for intensive care unit (ICU) patients (Teasdale & Jennett, 1974; Balestreri et al., 2004; Alvarez, Nava, Rué, & Quintana, 1998). Acute physiology and chronic health evaluation II (APACHE II) and Glasgow Coma Scale (GCS) are two systems currently in common use for measuring the condition of individual ICU patients (Chesnut, 1998).

Patients who are admitted to the neurosurgical ICU are likely in many instances to have higher mortality despite multimodal intensive management, regardless of their neurosurgical diagnosis (Walther, Jonasson, & Gill, 2003). The revised Acute Physiology and Chronic Health Evaluation system (APACHE II) has been frequently applied in many intensive care units (ICUs) throughout the world since 1985 (Knaus, Draper, Wagner, & Zimmerman, 1985; Cho, Wang, & Lee, 1995; Lane, Báez, Brabson, Burmeister, & Kelly, 2002; Hartley, Cozens, Mendelow, & Stevenson, 1995).

APACHE II is a physiologically based system including 12 physiological parameters and it also includes GCS. APACHE II has been designated as an exact predictor of outcome across a wide range of diagnostic groups, but it has yet to gain wide acceptance in neurosurgery, where instead the GCS has been the standard against which other grading systems are compared. The system is thought to be superior to GCS due to its recognition of age and significant underlying problems (Caronna & Stübgen, 2000).

This study compares GCS and APACHE II scores in patients with multiple trauma in terms of prediction of efficiency and mortality.

## 2. Materials & Methods

This study included the consecutive patients with head trauma associated with systemic trauma between 2007 and 2008. The data were collected and analyzed to calculate APACHE II (table 1) and GCS scores (table 2) at the time of admission to the ICU. The values were further analyzed according to age, sex, organ trauma, and treatment protocol.

**Table 1:** The items of APACHE II system

Physiological Variable	High Abnormal Range					Low Abnormal Range				

	+4	+3	+2	+1	0	+1	+2	+3	+4	Points
Temperature	≥41°	39 to 40.9°		38.5 to 38.9°	36 to 38.4°	34 to 35.9°	32 to 33.9°	30 to 31.9°	≤29.9°	
Mean Arterial Pressure-mm Hg	≥160	130 to 159	110 to 129		70 to 109		50 to 69		≤49	
Heart Rate	≥180	140 to 179	110 to 139		70 to 109		55 to 69	40 to 54	≤39	
Respiratory Rate	≥50	35 to 49		25 to 34	12 to 24	10 to 11	6 to 9		≤5	
Oxygenation: A-aDO_2_ or PaO_2_ (mm Hg)	≥500	350 to 499	200 to 349		<200					
a. FD_2_ ≥0.5 record A-aD0_2_										
b. FD_2_ >0.5 record PaO_2_					PO_2_>70	PO_2_ 61 to 70		PO_2_ 55 to 60	PO_2_<55	
Arterial pH	≥7.7	7.6 to 7.69		7.5 to 7.59	7.33 to 7.49		7.25 to 7.32	7.15 to 7.24	<7.15	
Serum HCO_3_ (venous mEq/l)	≥52	41 to 51.9		32 to 40.9	22 to 31.9		18 to 21.9	15 to 17.9	<5	
Serum Sodium (mEq/l)	≥180	160 to 179	155 to 159	150 to 154	130 to 149		120 to 129	111 to 119	≤110	
Serum Potassium (mEq/l)	≥7	6 to 6.9		5.5 to 5.9	3.5 to 5.4	3 to 3.4	2.5 to 2.9		<2.5	
Serum Creatinine (mEq/l)	≥3.5	2 to 3.4	1.5 to 1.9		0.6 to 1.4		<0.6			
Haematocrit (%)	≥60		50 to 59.9	46 to 49.9	30 to 45.9		20 to 29.9		<20	
White Blood Count (total/mm^3^)	≥40		20 to 39.9	15 to 19.9	3 to 14.9		1 to 2.9		<1	
Glasgow Coma Score (GCS)										
Score = 15 minus actual GCS										

A. Total Acute Physiology Score (APS) (sum of 12 above points) B. Age points (years) ≤44=0; 45 to 54=2; 55 to 64=3; 65 to 74=5; ≥75=6 C. Chronic Health Points (see below) Total APACHE II Score (add together the points from A+B+C

**Table 2:** GCS components

Eye opening	Best verbal response	Best motor response
		6: obey commands
	5: oriented	5: localizes
4: spontaneous	4: confused	4: withdraws
3: to speech	3: inappropriate words	3: abnormal flexion
2: to pain	2: incomprehensible sounds	2: extension

1: none	1: none	1: none
Total GCS Score: 3-15		

For the APACHE II calculation, physiological variables were obtained within the first 24 hours of admission to the intensive care unit, as were the age and information on chronic disease. On the other hand, an equation established by Knaus et al. in 1985 was used for the calculation of mortality risk. In the case of sedated patients still under immediate post-anesthesia observation, the score relating to the assessment of consciousness level via GCS was calculated only after the patient had overcome the anesthetic effect. For intubated patients, this score was calculated considering their capacity to understand, regardless of speech. Recorded and expected mortality rates were compared for each group of patients, and the standard mortality ratio was calculated.

Student’s t test was used for comparing the averages of continuous measurements. The chi-squared test was used for comparing the proportions of categorized measurements and showing trends in situations of ranking. Averages across more than two groups were compared via analysis of variance between groups (ANOVA).

A Receiver Operating characteristic (ROC) curve depicted the relation between true positive (sensitivity or number of predicted deaths/number of deaths) and false positive (1-specificity, or number of predicted deaths/number of survivors) for each scale. The area under the ROC curve was evaluated. A value of 0.5 under the ROC curve indicated that the variable performs no better than chance, while a value of 1.0 indicates perfect discrimination. A larger area under the ROC curve represents more reliability and good discrimination of the scoring system. The predictive capability of the index was assessed using the receiver operating characteristic curve, through a 2 × 2 decision matrix and linear regression analysis. The Statistical Package for the Social Sciences (SPSS) program were used and p < 0.05 was considered statistically significant.

## 3. Results

The study included 93 patients (79 males, 14 females). Mean age was 60.5 with the range from 14 to 87 years. In our cases 71.5% patients were admitted due to motor vehicle accident, 23.2% due to fall and 5.3% with a street battle. The mortality in relation to different APACHE II (table 3) and GCS (table 4) were evaluated. The patients were analyzed according to age, sex, and details of systemic trauma. Although there was no significant difference for sex in terms of mortality (p=0.459), mortality increased in the elderly group (p<0.001). This result supports the fact that APACHE II could be better than GCS in predicting mortality.

**Table 3:** GCS of Patients and mortality in ICU

GCS	Number/Percent (%)	Mortality/Percent (%)
13-15	18 (19.4)	3 (3.2)
9-12	27 (29.0)	6 (6.4)
3-8	48 (51.6)	9 (9.7)
**Total**	93 (100)	18 (19.3)

**Table 4:** APACHE II score of patients and mortality in ICU

APACHE II Score	Number/Percent (%)	Mortality/Percent (%)
0-5	5 (5.4)	0 (0)
6-10	19 (20.3)	1(1.1)
11-15	22 (23.7)	2(2.1)
16-20	20 (21.5)	2(2.1)
21-25	18 (19.4)	3(3.2)
26-30	5 (5.4)	4(4.4)
>30	4 (4.3)	6(6.4)

**Total**	93 (100)	18/(19.3)

All of the cases had multiple trauma. Patients with orthopedic problems formed the largest group (35.3%). Other groups were categorized in a decreasing order of frequency as follows: craniofacial trauma 24.4%, thoracic trauma 21.8%, spinal trauma 13.2% and abdominal trauma 5.3%.

**Figure 1:**
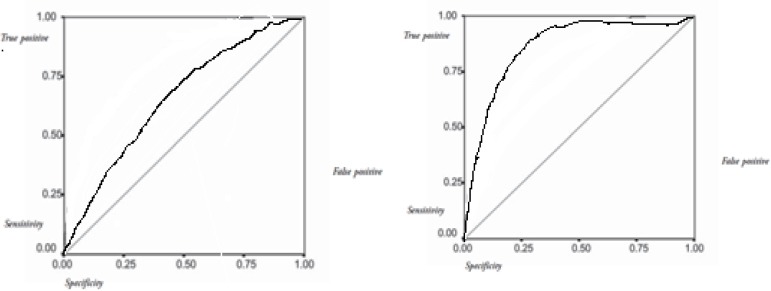
The receiver operative characteristic curves for APACHE II score (Right) and GCS (Left)

18 patients died in hospital, which indicates a mortality ratio of 19.5% in this study. The mortality was higher in groups with thoracic and orthopedic trauma but there was no statistical influence of associated trauma over mortality. Surgical treatment was performed in 35.3% due to head or systemic trauma. The mean survival score using APACHE II was 36.5 and death score was 67.4 (p<0.001). These values using GCS were 10.3 and 6.8, respectively (p<0.001) (table 5). The area under the ROC curve was larger in APACHE II (0.892±0.028) than GCS (0.621±0.029) (figure 1).

**Table 5:** The mean survival and death scores

Score	APACHE II Median point	GCS Median point
**Death**	67.4	6.8
**Survival**	36.5	10.3

## 4. Discussion

The GCS is a physiological scoring system, and it remains a critical measure of neurological assessment and assessment of severity of traumatic head injury on admission.

It is known that a low GCS is associated with poor prognosis; however, the measurement can be complicated in some cases. This scale measurement can be difficult to assess when the patient is intubated, sedated, intoxicated or has maxillofacial injuries (Meredith, Rutledge, Fakhry, Emery, & Kromhout-Schiro, 1998; Livingston, Mackenzie, MacKirdy, & Howie, 2000). Furthermore, the age factor, which is not included in GCS.

Systemic hypotension, intracranial hypertension, arterial hypoxia, and hypocapnia are known to be associated with poor outcome after head injury.

APACHE II contains 12 physiologic variables that are useful predictors of hospital outcome in ICU patients. (Knaus, Draper, Wagner, & Zimmerman, 1985; Vassar, Lewis, Chambers et al, 1999). Moreover, it covers GCS, age and chronic health conditions, which are thought to reflect physiological reserve defined for regulation of homeostasis. Sacco et al. (Sacco, Copes, Bain, MacKenzie, Frey, Hoyt, et al., 1993) reported that premorbid chronic health status is included in APACHE II, and Milzman et al. (Milzman, Boulanger, Rodriguez, Soderstrom, Mitchell, & Magnant, 1992) concluded that pre traumatic medical status or organ dysfunction had a significantly adverse effect on survival of trauma patients. The inclusion of chronic health status can improve the prediction of outcome in ICU trauma patients (Cho, Wang, Lee, 1995) Superior prognostic results were reported among cases having both occlusive cerebrovascular and coronary diseases, (Wong, Barrow, Gomez, McGuire, 1996) when APACHE II was performed. Pathophysiological changes predicted in an organism after systemic trauma could be demonstrated easily by the APACHE II scoring system. However, there are several limitations of the APACHE II scoring system. For example, APACHE II classifies trauma patients into two separate groups of patients as head trauma and multiple trauma, or postoperative and non-operated groups. For the assessment of early mortality, GCS score still provides simple, less-time consuming and effective information concerning head injury patients, especially in an emergency situation; however, for the prediction of mortality, APACHE II is better than GCS as it additionally includes the main physiologic parameters of the patient.
